# Association of epicardial fat thickness assessed by echocardiography with the severity of coronary artery disease

**DOI:** 10.34172/jcvtr.2020.19

**Published:** 2020-05-31

**Authors:** Alireza Rostamzadeh, Kamal Khademvatani, Mir Hossein Seyed Mohammadzadeh, Shahrzad Ashori, Mojgan Hajahmadi Poorrafsanjani, Behzad Rahimi, Behshid Ghadrdoost

**Affiliations:** ^1^Seyyed-al Shohada University Hospital, Urmia University of Medical Sciences, Urmia, Iran; ^2^Urmia University of Medical Sciences, Urmia, Iran; ^3^Rajaie Cardiovascular Medical and Research Center, Iran University of Medical Sciences, Tehran, Iran

**Keywords:** Epicardial Fat Thickness, Coronary Artery Disease, SYNTAX Score, Echocardiography

## Abstract

***Introduction:*** Epicardial fat thickness (EFT) can reflect risk of cardiovascular disease particularly coronary artery disease (CAD). The aim of this study was to investigate the association of EFT assessed by echocardiography and presence as well as severity of CAD.

***Methods:*** Two hundred and twenty consecutive patients who candidate for coronary angiography because of possible CAD were studied. EFT was evaluated in standard parasternal long axis (PlAX) and parasternal short axis (PSAX) view from 3 cardiac cycles at the end of systole and diastole. The severity of CAD was defined in two ways: (1) SYNTAX score, (2) number of vessels with significant lesion.

***Results:*** PLAX (EFT_S_) (EFT in systole) and PLAX (EFT_d_) (EFT in diastole) were significantly higher in patients with CAD in comparison with patients without CAD (*P* = 0.046, *P* = 0.041 respectively). There was a significant correlation between PLAX (EFT_S_) (*P* = 0.05), PLAX (EFT_d_) (*P* = 0.04) and SYNTAX score. There was no statistically significant relationship between EFT and number of diseased vessel (*P* > 0.05). Multivariate analysis was done for adjusting the effects of confounding factors and it showed that EFT (OR: 10.53, *P* = 0.004) was significantly correlated severe CAD as assessed by the SYNTAX score.

***Conclusion:*** EFT assessed by transthoracic echocardiography was higher significantly in patients with CAD than in normal patients. EFT as an easily available and cost-effective echocardiographic feature might be useful to predict complexity of CAD.

## Introduction

Internal organs are restricted by adipose tissue. In heart, adipose tissue can be subdivided into intra and extra pericardial fat that is located between the myocardium and visceral pericardium, particularly around subepicardial coronary vessels.^1,2^ In physiologic situations, epicardial fat tissue consists 20% of the heart’s weight which is mostly deposited in the atrioventricular and interventricular grooves, exactly where the coronary arteries are located that seems to be potential interactions between epicardial fat and myocardium.^1,3^

Epicardial fat is necessary for adequate cardiac function because it has an essential role in preserving the equivalence of body energy and metabolic process, but in other hand, it has important impact on heart muscle function because of secretion of adipokines and pre-inflammatory cytokines.^4^ It seems that increased thickness of epicardial fat increases the risk of developing of metabolic syndrome which itself causes coronary artery disease (CAD).^5^ This is likely several cytokines which play a role in the formation of vascular plaques and protein related to atherosclerosis are be expressed by epicardial adipose tissue.^6^ It seems that regional body adipose tissue is better and stronger marker of cardiovascular disease than total body adiposity. So, fat deposited around the heart and coronary arteries, can lead us to stratify the risk of cardiovascular disease particularly CAD and its severity.^7^

Epicardial fat thicknesses (EFTs) can be quantified by echocardiography as a reliable imaging marker and less expensive modality than MRI or CT.^8^ Some studies showed the relationship between EFT and severity of CAD with conflicting results. Therefore, the purpose of current study is to evaluate the association between EFT, evaluated by transthoracic echocardiography, and severity of CAD and find the related factors.

## Materials and Methods

### Study population

In this cross sectional study, 220 consecutive patients who underwent coronary angiography because of suspected CAD were studied. Patients with normal thoracic shape, sinus rhythm were included. Patients who had history of revascularization, cardiomyopathies, severe valvular heart disease and non-sinus rhythm were not included in the study. Patients who had inadequate transthoracic echocardiographic imaging for the measurement of EFT were excluded.

### Anthropometric and laboratory assessment

All patients underwent anthropometric measurement including height and weight for calculating body mass index (BMI) and hip and waist circumference for calculating waist/hip ratio. Routine biochemistry test including fasting blood sugar, HbA1C and lipid profiles (total cholesterol, low-density lipoprotein cholesterol (LDL-C), high-density lipoprotein cholesterol, and triglyceride (TG) levels) was checked in all patients in the morning after overnight fasting.

Regard to strong power of EFT to predict CAD in patients with BMI<27 kg/m^2^, we divided patients to two group; BMI<27 kg/m^2^ and BMI ≥27 kg/m^2^.^9^

Hypertension was defined as systolic blood pressure (SBP) of 140 mm Hg or more, or a diastolic blood pressure (DBP) of 90 mm Hg or requirement for antihypertensive medication. Diabetes mellitus was defined as hyperglycemic conditions that require insulin or oral hypoglycemic drugs according to the criteria of the American Diabetes Association. Smoker was defined someone who has smoked regularly in their lifetime and has smoked in the last 28 days. Hyperlipidemia was defined as total cholesterol higher than 220 mg/d or triglycerides ≥150 mg/dL.^1^

### CAD definition and SYNTAX score calculation

Coronary angiographic analysis was performed by one expert cardiologist who was unaware of the patients’ clinical information. Angiographic data were analyzed for the severity of CAD. The severity of CAD was defined in two ways: number of diseased vessel with significant lesion and based on SYNTAX score.

Severity of CAD base on the number of diseased vessel was divided to 5 groups: normal coronary artery or mild CAD (angiographically coronary artery lesions <40% lumen diameter stenosis),^10^ two vessel diseases, three vessel diseases and left main.

For assessing complexity of coronary stenosis, SYNTAX score was calculated for each patient by two experienced cardiologists who were blinded to patient’s clinical data. In case of divergence, the final conclusion was made by the opinion of the third observer. SYNTAX score is the sum of the points of individual lesion in the coronary tree which has more than 50% diameter narrowing in vessels >1.5 mm in diameter.^11^

### Echocardiography and EFT measurement

Transthoracic echocardiography for measuring EFT was done a day after coronary angiography with the Siemens Acuson (SC2000) instrument, using standard techniques, with subjects in the left lateral decubitus position. Offline measurements of EFT were performed by two echocardiography specialists, who were blind to clinical data and to the results of each other.

We measured EFT on the free wall of right ventricle along the midline of the ultrasound beam, perpendicular to the aortic annulus from the parasternal long-axis view at end-diastole for 3 cardiac cycles on the 2-dimensional echocardiography.

Epicardial fat was measured in standard parasternal long axis (PlAX) and parasternal short axis (PSAX) view from 3 cardiac cycles at the end of systole and diastole.

### Statistics

Numeric variables were expressed as mean values ± standard deviation and nominal and categorical variables were expresses as interval and count (%). All variables were tested for normal distribution with the Kolmogorov-Smirnov test. Student t-test was used for statistical comparisons of continuous variables between CAD and Non-CAD. To assess relationship between cutoff point of EFT and severity of CAD based on SYNTAX student t-test was used. The effect of EFT on severity of CAD was assessed using ANOVA test. Pearson correlation analysis was used for analysis of correlation between EFT and SYNTAX score. The ROC curve was used to determine the area under the curve (AUC), and the optimum EFT cut-off level to predict severity of CAD. Multivariate analysis was performed for prediction of the severe CAD measured by SYNTAX score. Statistical analyses were performed with SPSS (version 15; SPSS Inc. Chicago, Illinois).

## Results

The mean age of 220 patients was 59.2 ± 11.1 years, and 116 of the patients were male (52.7%). Anthropometrics, cardiovascular risk factors, laboratory data of enrolled patients are described in Table 1. The mean of epicardial fat in systolic and diastolic PlAX and PSAX view are also shown in Table 1.

**Table 1:** Baseline and clinical characteristics of patients

**Variables**	**Mean ± SD, No. (%)**
Age (y)	59.2±11.1
Sex (M/F)	116/104
Anthropometrics	
BMI	28.01±4.69
Waist circumference (cm)	97.35±9.07
Hip circumference (cm)	108.09±9.55
Waist/hip	0.90±0.05
Risk factors	
DM	143(65%)
HTN	96(43.6%)
DLP	167 (75.9%)
Smoking	164(74.5%)
Lab tests	
FBS (mg/dL)	121.09±53.24
HDL-H (mg/dL)	39.01±8.84
LDL-C (mg/dL)	88.19±30.64
Chol (mg/dL)	155.36±43.43
TG (mg/dL)	147.50±106.46
HbA1C	6.26±1.62
Epicardial thickness	
PLAX (EFTs) (cm)	0.61±0.20
PSAX (EFTs) (cm)	0.57±0.18
PLAX (EFT_d_) (cm)	0.35±0.15
PSAX (EFT_d_) (cm)	0.36±0.14

M: male, F: female, BMI: body mass index, DM: diabetes mellitus, HTN: hypertension, DLP: dyslipidemia, FBS: fasting blood sugar, HDL: high density lipoprotein-cholesterol, LDL: low density lipoprotein-cholesterol, Chol: cholesterol, TG: triglyceride, HbA1C: hemoglobin A1C, EFT: epicardial fat thickness

### Bivariate analysis

PLAX (EFTs) and PLAX (EFT_d_) were significantly higher in patients with CAD than in comparison with patients without CAD (*P* = 0.046, *P* = 0.041 respectively) (Table 2).

**Table 2:** Comparison of EFT between the CAD and non-CAD patients

	**CAD**	**None-CAD**	***P*** **value**
PLAX (EFT_S_) (cm)	0.62±0.20	0.55±0.21	0.046
PSAX (EFTs) (cm)	0.58±0.17	0.54±0.20	0.200
PLAX (EFT_d_) (cm)	0.36±0.15	0.31±0.12	0.041
PSAX (EFT_d_) (cm)	0.36±0.14	0.35±0.13	0.557

CAD, coronary artery disease; EFT, epicardial fat thickness.

In terms of the severity of CAD based on the score SYNTAX, there was a significant correlation between PLAX (EFT_S_) (*P* = 0.05), PLAX (EFT_d_) (*P* = 0.04) and SYNTAX score (Table 3).

**Table 3:** Correlation between EFT and SYNTAX score

**Variables**	**SYNTAX score**	
	**r**	***P*** **value**
PLAX (EFT_S_) (cm)	0.273	0.05
PSAX (EFTs) (cm)	0.111	0.71
PLAX (EFT_d_) (cm)	0.286	0.04
PSAX (EFT_d_) (cm)	0.070	0.64

EFT, Epicardial fat thickness.

CAD was also divided base on the number of diseased vessel to 5 groups: normal coronary artery or mild CAD, one vessel, two vessel diseases, three vessel diseases and left main. There was no statistically significant relationship between EFT and number of diseased vessel (*P* > 0.05) (Table 4).

**Table 4:** Association between EFT and number of diseased vessel

	**NCA or mild CAD**	**1 VD**	**2VD**	**3VD**	**LM**	***P*** **value**
PLAX (EFT_S_) (cm)	0.55±0.21	0.59±0.21	0.64±0.19	0.61±0.20	0.64±0.19	0.35
PSAX (EFTs) (cm)	0.54±0.20	0.59±0.21	0.60±0.15	0.59±0.20	0.57±0.17	0.56
PLAX (EFT_d_) (cm)	0.31±0.12	0.35±0.16	0.34±0.11	0.38±0.17	0.37±0.15	0.25
PSAX (EFT_d_) (cm)	0.35±0.13	0.34±0.12	0.34±0.12	0.39±0.18	0.36±0.21	0.36

NECA: normal coronary artery, 1VD: single vessel disease, 2 VD: two vessel disease, 3VD: three vessel disease, LM: left main, EFT: epicardial fat thickness.

Interestingly, there was a significant correlation between PLAX (EFT_S_) (*P* = 0.04), PSAX (EFT_S_) (*P* = 0.004), PLAX (EFT_d_) (*P* = 0.009) and SYNTAX score in patients with BMI<27 kg/m^2^(Table 5).

**Table 5:** Correlation between EFT and SYNTAX score in patients with BMI <27 kg/m^2^

**Variables**	**SYNTAX score**	
	**r**	***P*** **value**
PLAX (EFT_S_) (cm)	0.195	0.042
PSAX (EFT_s_) (cm)	0.276	0.004
PLAX (EFT_d_) (cm)	0.249	0.009
PSAX (EFT_d_) (cm)	0.152	0.115

EFT, epicardial fat thickness; BMI, body mass index.

### Multivariate analysis

Multivariate analysis was done for adjusting the effects of confounding factors and it showed that EFT (OR: 10.53, *P* = 0.004) was significantly correlated with severe CAD as assessed by the SYNTAX score (Table 6).

**Table 6:** Multiple regression analysis for prediction of the severe coronary artery disease measured by SYNTAX score

**Variable**	**Unstandardized coefficients**	**B standard error**	***P*** **value**	**Odds ratio**	**95% CI (Lower-Upper)**
EFT	4.24	0.30	0.04	10.53	3.502-48.212
Age	0.24	0.018	0.15	1.153	0.887-1.444
BMI	0.025	0.014	0.17	0.50	0.185-1.366
DM	0.031	0.114	0.652	15.256	0.199-1.123
DLP	0.034	-0.040	0.235	1.007	0.919-1.014
Smoking	0.035	-0.009	0.793	1.591	0.55-10.87
Sex	0.056	0.082	0.142	1.327	1.007-2.246

EFT: epicardial fat thickness, BMI: body mass index, DM: diabetes mellitus, DLP: dyslipidemia.

### Cut of points of EFS variables

To find the cutoff point for EFT, ROC curve was used. Based on the results of the ROC curve, for the existence of CAD based on epicardial fat thickness PLAX (EFT_S_), the best cut point of 0.51 cm was calculated. The sensitivity and specificity of this point for CAD is 66.7% and 65.7%, respectively. For PSAX (EFT_S_) and PLAX (EFT_d_), no significant cutoff points were calculated. The ROC Curve results are presented in Table 7. In our findings, there were a significant relationship between cutoff point of EFT and severity of CAD based on SYNTAX (Table 8). As shown in table 8, SYNTAX score was significantly higher in patients with PLAX (EFT_S_)**≥**0.51 (*P* = 0.04) and PSAX (EFT_S_) ≥0.47 (*P* = 0.01).

**Table 7:** ROC curve results for epicardial fat thickness

	**PLAX (EFT**_S_ **)**	**PSAX (EFT**_s_ **)**	**PLAX ( EFT**_d_ **)**
Area under curve	0.584	0.817	0.742
*P* value	0.038	0.058	0.066
Cut off point	0.51	0.47	0.41
95% CI	0.241-0.937	0.590-1	0.401-1
Sensitivity	66.7	100	100
Specificity	65.7	64.2	48.2

EFT, epicardial fat thickness.

**Table 8:** Relationship between epicardial fat thickness and severity of coronary artery disease based on SYNTAX score

		**M±SD**	***P*** **value**	**95% CI**	
				**Lower**	**Upper**
PLAX (EFT_S_) (cm)	<0.51	14.63±8.04	0.04	-7.01	-0.65
	≥0.51	18.17±9.76			
PSAX (EFTs) (cm)	<0.47	13.93±8.42	0.017	-7.92-	-0.80
	≥0.47	18.29±9.50			
PLAX (EFT_d_) (cm)	<0.41	16.11±9.14	0.10	-6.09	0.57
	≥0.41	18.87±9.66			

EFT, epicardial fat thickness.

## Discussion

The relationship between echocardiographic EFT and CAD has also been reported in several studies like our study.^8,12-14^ The present study was intended to show association between echocardiographic EFT and severity of CAD in patients with CAD.

In this study, 220 patients who candidate for coronary angiography underwent to echocardiography and EFT in PSAX and PLAX view at the end of systole and diastole phase was measured. For evaluation the association between EFT and severity of CAD, the number of vessel with significant stenosis and SYNTAX score was used. Cut off point of PLAX (EFTs), PSAX (EFTs) and PLAX (EFT_d_) for CAD was obtained.

Parameters that are easily measurable as well as reliable are needed to estimate CAD presence and severity. Considering that various studies have used different methods for determining the severity of CAD and its relation with EFT we decided to use a combination of different methods. For this purpose, the severity of CAD was evaluated by SYNTAX score and the number of coronary diseased vessel.

SYNTAX score as an angiographic scoring system represents the coronary obstructive lesions complexity and associates with risk of major adverse cardiac events in patients with CAD.^15^ SYNTAX score is used to estimate the complexity of the CAD through the assessment of the number of diseased coronary arteries, their functional effects, locations, and complexity detected by angiography.^16^

There are some scoring systems to determine the severity of the CAD such as Gensini score that are used to evaluate the relationship between CAD complexity and EFT. Jeong et al showed the relationship between echocardiographic EFT and severity of CAD but in that study, severity of CAD was evaluated by Gensini score. They concluded that the patients with a higher EFT were associated with a high Gensini score.^17^

In our study, SYNTAX scoring system in parasternal long axis view at the end of systolic and diastolic phase had significant relationship with EFT. It should also be considered that some parameters that have impact on the SYNTAX score such as right or left dominancy, vessel tortuosity, bifurcation lesions, age, might not have a relationship with atherosclerosis directly. In current study, we found a significant relationship between SYNTAX score and EFT in all patients and in patients who had BMI less than 27 kg/m^2^ means in patients with a non-high BMI. We used cut-off point of 27 kg/m^2^ based on the percentage of body fat in the Asian population while in western population BMI ≥30 kg/m^2^ is considered as cut-off point for obesity.^16^

A study showed increased EFT volume were associated with CAD and extensive coronary artery calcium measured by CT in patients with BMI < 27 kg/m^2^ however we used echocardiography to measure EFT instead of CT.^18^

Some possible reasons can be considered to justify a stronger relationship between EFT and severity of CAD in patients with BMI <27 kg/m^2^ than patents with BMI ≥27 kg/m^2^: in patients with high BMI group, total amount of visceral adipose tissue is high and the EFT might be a small proportion of it. So in patients with BMI ≥27 kg/m^2^ EFT is not a precise indicator to predict the severity of CAD in the high BMI group^16^ and also in patients with high BMI, adipokines that stimulate inflammatory responses and upregulate in obesity, have been promote obesity-induced cardiovascular diseases and it does not show correctly the correlation with complexity of CAD.^16,19^

Even though there were no statistically significant relationship between traditional risk factors of CAD such as age, male sex, diabetes, smoking, BMI, and dyslipidemia, the correlation between EFT and severe CAD calculated by SYNTAX score was significant. It is worth noting that this relationship was not found when the severe CAD was defined based on the number of diseased vessel. It might be because SYNTAX score is more efficient and precise than number of diseased vessel in estimation of severity of CAD because The SYNTAX score consists of several valid angiographic parameters used to classify coronary artery lesions due to their functional impact, location, and severity.^20^

In our study, PLAX (EATs) was more precise and reliable index than others which could even diagnose stenosis less than 50% CAD sufficiently.

In current study, EFT was measured by 4 methods that showed different outcomes. Generally, we found that although there are significant relationship between EFT and severity of CAD, more studies are needed to evaluate the factors which are influence on the thickness of epicardial fat and also in different group of patients who take benefits from EFT measurement as a screening method.

## Conclusion

In this study, EFT assessed by transthoracic echocardiography was higher significantly in patients with CAD than in normal patients. So EFT might be useful to predict complexity of CAD, especially one less than 27 kg/m^2^. These findings may show the importance of echocardiography as a reliable and non-expensive assessment tool to predict prognosis in patients with CAD.

## Competing interests

None.

## Ethical approval

This study was approved by the ethical committee of Urmia University of Medical Sciences according to the Helsinki Declaration of the World Medical Association (2000). All patients were informed and given written consent form (Ethics No. IR.UMSU.RES.1395.99).

## Funding

This research did not receive any specific grant from funding agencies in the public, commercial, or not-for-profit sectors.

## Acknowledgments

The authors would like to thank the colleagues in Seyyed-al Shohada University Hospital, Urmia University of medical sciences.
